# VERDICT MRI validation in fresh and fixed prostate specimens using patient‐specific moulds for histological and MR alignment

**DOI:** 10.1002/nbm.4073

**Published:** 2019-02-19

**Authors:** Colleen Bailey, Roger M. Bourne, Bernard Siow, Edward W. Johnston, Mrishta Brizmohun Appayya, Hayley Pye, Susan Heavey, Thomy Mertzanidou, Hayley Whitaker, Alex Freeman, Dominic Patel, Greg L. Shaw, Ashwin Sridhar, David J. Hawkes, Shonit Punwani, Daniel C. Alexander, Eleftheria Panagiotaki

**Affiliations:** ^1^ Centre for Medical Image Computing University College London London UK; ^2^ Sunnybrook Research Institute Toronto ON Canada; ^3^ Discipline of Medical Radiation Sciences The University of Sydney Sydney Australia; ^4^ Centre for Advanced Biomedical Imaging University College London London UK; ^5^ Imaging Francis Crick Institute London UK; ^6^ Centre for Medical Imaging University College London London UK; ^7^ Division of Surgery and Interventional Science University College London London UK; ^8^ Department of Urology University College London Hospitals London UK; ^9^ Department of Research Pathology University College London London UK

**Keywords:** cell density, diffusion MRI, histological validation, prostate cancer, VERDICT

## Abstract

The VERDICT framework for modelling diffusion MRI data aims to relate parameters from a biophysical model to histological features used for tumour grading in prostate cancer. Validation of the VERDICT model is necessary for clinical use. This study compared VERDICT parameters obtained ex vivo with histology in five specimens from radical prostatectomy. A patient‐specific 3D‐printed mould was used to investigate the effects of fixation on VERDICT parameters and to aid registration to histology. A rich diffusion data set was acquired in each ex vivo prostate before and after fixation. At both time points, data were best described by a two‐compartment model: the model assumes that an anisotropic tensor compartment represents the extracellular space and a restricted sphere compartment models the intracellular space. The effect of fixation on model parameters associated with tissue microstructure was small. The patient‐specific mould minimized tissue deformations and co‐localized slices, so that rigid registration of MRI to histology images allowed region‐based comparison with histology. The VERDICT estimate of the intracellular volume fraction corresponded to histological indicators of cellular fraction, including high values in tumour regions. The average sphere radius from VERDICT, representing the average cell size, was relatively uniform across samples. The primary diffusion direction from the extracellular compartment of the VERDICT model aligned with collagen fibre patterns in the stroma obtained by structure tensor analysis. This confirmed the biophysical relationship between ex vivo VERDICT parameters and tissue microstructure from histology.

**Abbreviations used**
ADC: apparent diffusion coefficient
AIC: Akaike information criterion
FA: fractional anisotropy
FOV: field of view
FWHM: full width at half maximum
H&E: haematoxylin and eosin
LWI: luminal water imaging
NODDI: neurite orientation distribution and density imaging
ROI: region of interest
SNR: signal‐to‐noise ratio
*T*_E_: echo time
*T*_R_: repetition time
VERDICT: vascular extracellular and restricted diffusion for cytometry in tumours

## 1 INTRODUCTION

Treatment decisions in prostate cancer are strongly influenced by tumour grade, which is determined by examination of cellular features and tissue microstructure in histological images. This histology is obtained using invasive biopsy procedures, which are problematic for screening large numbers of patients and longitudinal patient monitoring. A non‐invasive imaging method that captures microstructural information is therefore desirable.

Diffusion MRI is sensitive to changes in epithelium, lumen and stroma,^1^ but conventional methods summarize this information using a single parameter, the apparent diffusion coefficient (ADC), which has relatively low specificity for identifying prostate cancer.^2^, ^3^ The vascular extracellular and restricted diffusion for cytometry in tumours (VERDICT) model for prostate distinguishes benign from malignant tissue with greater accuracy than conventional diffusion metrics.^4^ VERDICT models the tissue with a simplified version of the histological features, with compartments describing the vascular, extravascular‐extracellular and cellular spaces.^5^ This provides biologically meaningful parameters related to vascular and cellular fractions, as well as an average cell size estimate. However, the biological interpretation of these parameters relies on the validity of the tissue model, which is a very simplified description of real complex tissue. VERDICT must therefore be validated against histology to determine the accuracy of the parameters that the model provides.

Recently, VERDICT parameters from ex vivo breast tissue were compared to histology,^6^ revealing an association between the intracellular fraction from MRI and the cell fraction from histology. The remaining signal component, assumed to be extracellular, was anisotropic, with directions similar to collagen fibre patterns in the stroma, but fibres were coherent only over distances of 1–2 mm, less than a typical clinical voxel size. An even stronger anisotropy has been observed in high‐resolution ex vivo studies of prostate^7^, ^8^, ^9^ and comparison of diffusion tensor parameters with histology suggests that high‐resolution diffusion anisotropy relates to stromal features, but this anisotropy is difficult to visualize at clinical voxel scales. Models of subvoxel variation in fibre direction are used routinely in brain imaging, for example the neurite orientation distribution and density imaging (NODDI),^10^ which assumes a Watson distribution of stick‐like structures that restrict diffusion to a single dimension. However, such models have not been tested in cancer settings despite the orientational heterogeneity observed in many cancer microenvironments.

VERDICT validation in prostate has been limited to histology from biopsy cores,^4^ which are difficult to spatially localize within the MRI volume. For validation, model parameters need to be compared with corresponding histological features (ie co‐registered images). The challenges of such a comparison may be divided into three stages: (1) those occurring as a result of surgery upon removal of the prostate from the body, including the loss of any functional information such as blood flow or tissue deterioration; (2) changes resulting from tissue fixation, which include the effects of fixation on water content, compartment size and shape, and (3) tissue changes as a result of histological processing and slicing.

We recently developed a 3D printing technique to construct a patient‐specific mould containing landmarks and slicing guides to manage several of these issues.^11^ In this paper, we address the questions associated primarily with the last two stages of the validation process. Diffusion‐weighted images were acquired before and after fixation in ex vivo prostatectomy samples and data were fitted using the VERDICT framework to examine changes. The resulting parameter maps were then compared to registered histological images sliced in the MR imaging plane to determine the validity of the ex vivo VERDICT parameters.

## 2 METHODS

### 2.1 Sample preparation

Five radical prostatectomy specimens (four Gleason 3 + 4, one Gleason 3 + 3) were prepared for ex vivo scanning as described previously.^11^ Briefly, in vivo images were acquired on a 3 T MRI (Philips Achieva, Best, The Netherlands) using a 3D *T*_2_‐weighted axial sequence (fast spin echo, *T*_E_ = 100 ms, *T*_R_ = 5.2 s, echo train length = 16, field of view (FOV) 18 cm × 18 cm, 0.4 × 0.4 × 2.5 mm^3^ resolution). The prostate was contoured on each slice by a radiologist (E.J., M.B., each with two years' experience) and the contours were formed into a 3D volume that was subtracted from a generic mould template in Rhino 5 (McNeel North America, Seattle, WA, USA). The mould was 3D printed from a nylon powder (PA2200 powder, 40–50 μm size) using selective laser sintering (EOSINT P100).

Seminal vesicles were removed before placing the specimen in the mould. The prostate was imaged fresh in saline (within 8 h of removal), then fixed in 10% formalin for at least 10 h, washed and placed in saline for at least 8 h and imaged a second time after fixation. Scanning temperature was 18.5 ± 0.5°C. Then a single 5 mm reference slice was cut before the prostate was removed from the mould for further histological processing. The porous grid design allowed for fixative penetration into the mould. Mould landmarks identifying a reference plane were located in MRI using gradient echo images spaced 0.5 mm apart, allowing co‐localization of the fresh and fixed images. Guides in the mould determined the histological slicing plane that corresponded to the reference plane, as described by Bourne et al.^11^

### 2.2 Ex vivo MRI data acquisition

Ex vivo images were acquired at 9.4 T (Agilent Inc., Santa Clara, CA, USA) using 400 mT/m gradients and a 72 mm internal diameter quadrature coil (RAPID Biomedical, Rimpar, Germany). A high‐resolution *T*_2_‐weighted (fast spin echo) sequence (repetition time *T*_R_ = 6 s, echo time *T*_E_ = 20 ms, 0.375 × 0.375 × 0.5 mm^3^ resolution, 96 × 96 mm^2^ FOV at 9.4 T, or *T*_R_ = 5.2 s, *T*_E_ = 100 ms, 0.4 × 0.4 × 2.5 mm^3^ resolution, 180 × 180 mm^2^ FOV at 3 T) was acquired for registration purposes. A multi‐echo *T*_2_ sequence (*T*_R_ = 2.2 s, *T*_E_ = 3 ms, 96 echoes, 1.25 × 1.25 × 2.5 mm^3^, 80 × 80 mm^2^ FOV) was also acquired to examine the nature of the *T*_2_ relaxation.

Diffusion protocols differed for fresh and fixed scans due to timing constraints, but both used a multi‐slice pulsed gradient spin echo for diffusion weighting, *T*_R_ = 2 s, and had the same geometry (1.25 × 1.25 mm^2^ in plane, eight 2.5 mm thick slices with 2.5 mm gap, 80 × 80 mm^2^ FOV). Diffusion‐weighted images (three orthogonal directions + one unweighted image) were acquired using a fast spin echo readout for fresh scans (diffusion parameters and echo times in white in Table 1) and a spin echo readout for fixed scans (parameters in grey in Table 1). In addition, a high‐angular‐resolution scan (*δ* = 4.5 ms, *Δ* = 20 ms, *T*_E_ = 36 ms, *G* = 18.7 mT/m, with 20 directions for the fresh protocol and 30 directions for fixed^12^) was acquired. The VERDICT parameters for fits to data from the two acquisition protocols were compared in one fixed tissue sample.

**Table 1:** Scan parameters for the fresh and fixed protocols. The value in each gradient separation (*Δ*) + duration (*δ*)/gradient strength (*G*) box corresponds to the *b*‐value (s/mm^2^) for that scan. Empty boxes indicate that gradient strength was not included in the protocol. Values in parentheses show the number of averages for a particular scan (no parentheses, one average)

*Δ*/*T*_E_ (ms)	*δ* (ms)		*G* (mT/m): 40	80	120	160	200	240	280	320	360	400
10/19	3	Fresh	9			148		334		594	752	
		Fixed	9		84		232		455		752	928
30/46	3	Fresh	30	120		478		1077 (2)				
		Fixed	30		269		748 (2)		1 465 (2)			
	10	Fresh	306	1222	2750		7638 (2)		14 971 (2)			
		Fixed	306		2750		7638 (2)		14 971 (2)			
50/66	3	Fresh	51	202		808 (2)						
		Fixed	51		455		1263 (4)					
	10	Fresh	535	2139		8555 (4)						
		Fixed	535		4812		13367 (4)					
70/86	3	Fixed	71		640 (2)		1779 (6)					
	10	Fixed	764		6875 (2)		19096 (6)					

### 2.3 Data analysis

The diffusion data were fitted to a range of compartmental models outlined in Table 2. The compartments are described mathematically in the Supplementary Material and References 10, 13, and include ball (isotropic, unrestricted diffusion), tensor (anisotropic diffusion described using three directions), sphere (isotropically restricted diffusion) and Watson (a group of ‘sticks’, each with diffusion restricted to a single dimension, having a Watson distribution of angles). Models combine compartments by summing the theoretical signal for compartments. All models include a normalization constant, *S*_0_, and a *T*_2_ relaxation time constant, *T*_2_.

**Table 2:** Compartmental models included in the model selection process, along with the number of free parameters fitted. All models include a normalization parameter, *S*_0_, and *T*_2_ to account for varying *T*_E_

Model	Num. Params
Ball (ADC)	3
Zeppelin	6
Tensor	8
Watson	6
Ball‐ball (bi‐exp)	4
Zeppelin‐ball	7
Tensor‐ball	9
Watson‐ball	7
Ball‐sphere	5
Zeppelin‐sphere	8
Tensor‐sphere	10
Watson‐sphere	8
Watson‐ball‐sphere	9*

* Watson stick and ball compartments in Watson‐ball‐sphere are considered to have the same diffusion coefficient.

Several one‐ and two‐compartment models were tested, including the conventional diffusion tensor (tensor) and bi‐exponential (ball‐ball) models. A separate ADC, ADC_*b* < 1000_, was calculated using data with *b*‐values under 1000 s/mm^2^ to imitate the output of current common clinical practice. Two‐compartment models with a spherical restricted compartment are based on VERDICT models applied in vivo, but without the third vascular compartment. In models with two compartments, the ball or sphere is assumed to represent the intracellular space and the diffusion coefficient was fixed to *D*_I_ = 0.3 × 10^−3^ mm^2^/s. This value was obtained by fixing *D*_I_ to a range of values between 0.1 and 1.5 and fitting data from five separate high‐SNR (signal‐to‐noise ratio) regions using the ball‐sphere and tensor‐sphere models. There was little variation in the objective function, but a minimum near 0.3 × 10^−3^ mm^2^/s was observed. This is in agreement with previous studies^14^ showing that the fit is insensitive to the value of *D*_I_, although very low values can affect the radius parameter.

Data were fitted voxelwise using a maximum‐likelihood approach that accounts for Rician noise.^13^ A noise estimate was calculated for each voxel using the standard deviation across repeated *b* = 0 images with *T*_E_ = 19 ms. Models were compared using the Akaike information criterion (AIC):

$$\mathrm{AIC}=2k-2\;\ln\left(L\right),$$

where *k* is the number of model parameters and *L* is the likelihood function for the model.

A conventional colour fractional anisotropy (FA) map was generated to display the primary diffusion direction and anisotropy. An additional in‐plane anisotropy map, which displays the primary diffusion direction within the imaging plane, was generated for comparison with the 2D histology. The 3D FA was used as the anisotropy measure in this in‐plane map and only the anisotropic compartment of the model was used (ie, in the tensor‐sphere model, only the anisotropy of the tensor is considered).

### 2.4 Histology and structure tensor analysis

Three micrometre whole mount sections of the prostate were cut at 5 mm spacing. The first 5 mm slice used the cutting guides in the mould to obtain a similar orientation to the MR imaging plane and a location near the centre for the MRI reference slice. Slices were stained with haematoxylin and eosin (H&E) and digitized at 20× objective magnification (Hamamatsu Nanozoomer, Hamamatsu City, Japan). Regions of low cellularity were identified by the low numbers of dark purple cell nuclei.

Structure tensor analysis^15^, ^16^ was performed on 5× magnification (1.8 μm resolution) images converted to greyscale. Analysis was restricted to the stroma, selected using *k*‐means clustering with three clusters (see Supplementary Figure 1). The structure tensor describes the local image orientation by convolving the image (*I*) with a 2D Gaussian weighting function (*w*) in a neighbourhood Ω:

$$J\left(x,y\right)=\iint_{\Omega \left(x,y\right)}w\left(\xi -x,\eta -y\right)\nabla I\left(\xi ,\eta \right)\nabla^{T}I\left(\xi ,\eta \right)\;d\xi \;d\eta .$$

A Gaussian with full width at half maximum (FWHM) of 15 μm was used, which is near the average distance a water molecule is expected to diffuse over a 30 ms MRI experiment. This value appeared to characterize the histological microstructure well compared with other FWHMs between 1 and 20 μm. An anisotropy index was calculated using the eigenvalues, *λ*_1_ and *λ*_2_, of the structure tensor: $\mathrm{AI}=\frac{\lambda_{1}-\lambda_{2}}{\lambda_{1}+\lambda_{2}}$. The eigenvector of the smaller eigenvalue gives the dominant direction for that pixel.

### 2.5 Registration

The *T*_2_‐weighted image from the reference slice of the fresh specimen was registered to the *T*_2_‐weighted image of the fixed specimen by a 2D rigid registration using a block‐matching strategy and correlation coefficient as similarity measure.^17^, ^18^

H&E images were downsampled to 0.25× and converted to greyscale for registration. Six corresponding points were chosen on the *T*_2_‐weighted fixed image and the corresponding histological slice. These points generated an initial transformation that was refined using a 2D rigid registration with block‐matching strategy and correlation coefficient as similarity measure. The final transformations were applied to the diffusion parameter maps, resampling using nearest‐neighbour interpolation to avoid discontinuities at boundaries; diffusion directions were also rotated using the transformations.

### 2.6 Statistics

For comparison of fresh and fixed parameters, regions of interest (ROIs) were drawn (by E.J. and M.B.) around the peripheral zone and transition zone in the reference slice. Tumour was also outlined when present in the reference slice (*n* = 2). Voxels from the fresh parameter maps were compared pairwise with voxels from the fixed parameter maps using the Wilcoxon signed‐rank test. Comparisons between parameter values across ROIs in the same specimen were made using the Mann–Whitney *U*‐test. Values were considered significant with *p* < 0.05/48 to correct for multiple comparisons.

## 3 RESULTS

**Figure 1:**
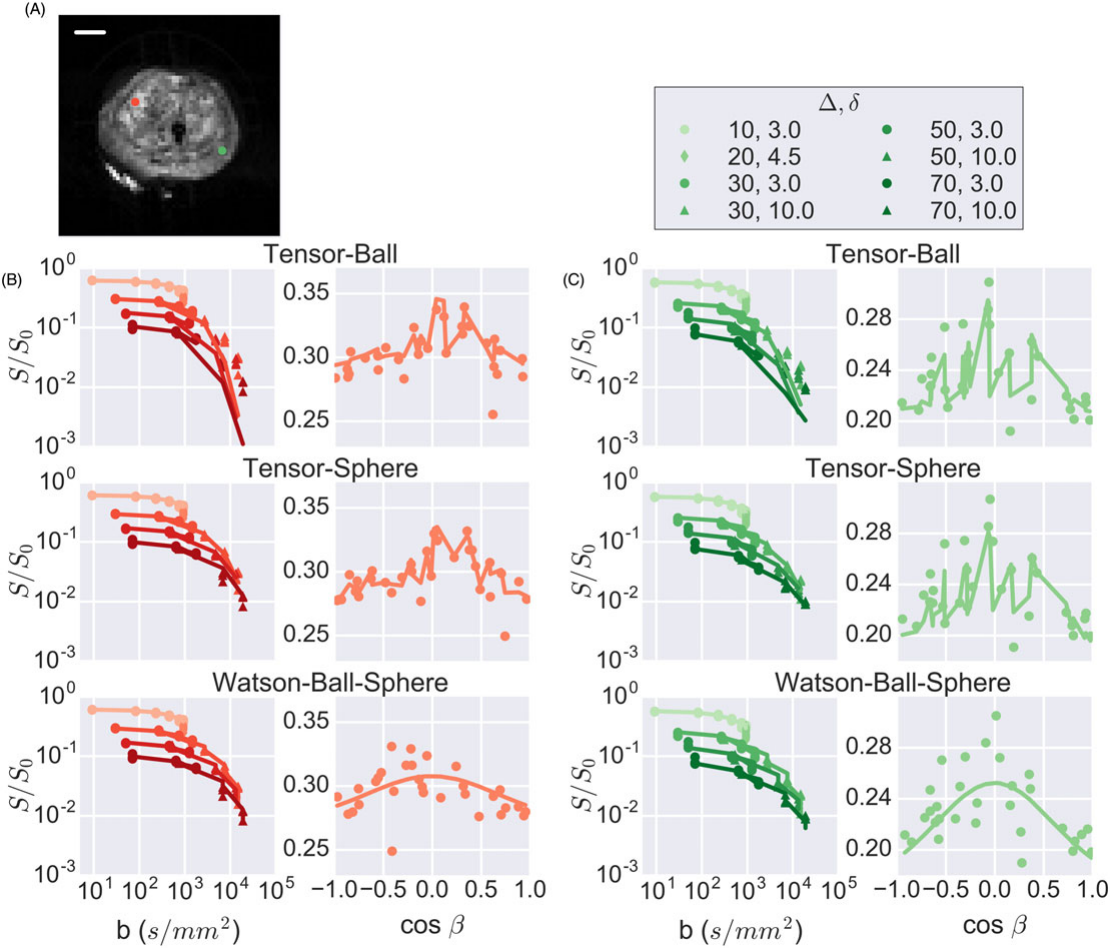
Model fitting results. A, diffusion‐weighted imaging indicating two points with high (red) and low (green) SNR. B, fits to the data from the high‐SNR voxel for three models (see supplementary figure 2 for remaining fits) indicating that tensor‐sphere and Watson‐ball‐sphere capture the effects of restriction at high *b*‐values while tensor‐ball and tensor‐sphere better capture the variation with gradient direction. Signal is plotted versus cos *β*, where *β* is the angle between the primary diffusion direction and the gradient direction. Plots for tensors are not smooth because the signal also depends on the secondary diffusion direction. C, fits for the lower‐SNR voxel show similar trends as in B, but with less deviation at high *b*‐values for the tensor‐ball model and more signal dependence on gradient direction. The scale bar is 10 mm

Figure 1A shows a diffusion‐weighted image (*b* = 1465 s/mm^2^). The image appears heterogeneous, with SNR of 81 for the red voxel and 19 for the green voxel. This difference was due partly to different initial signals (proton density and coil sensitivity effects) and partly to smaller diffusion signal attenuation in the red voxel. For the largest diffusion weighting, *b* = 19 096 s/mm^2^ with six signal averages, the SNR is 5.8 in the red voxel and 1.7 in the green voxel.

Figure 1B and 1 C shows representative results from the fitting. The VERDICT parameters for the ball‐sphere model calculated from data from the fresh and fixed protocols in fixed tissue agreed within 15% (data not shown) except in areas where the radius was poorly determined, discussed further below. The fits for the higher‐SNR voxel (Figure 1B) demonstrated that the tensor‐ball model was unable to capture the effects of restricted diffusion at high *b*‐values whereas the tensor‐sphere and Watson‐ball‐sphere models both captured these changes. The signal dependence on cos *β* is also plotted, where *β* is the angle between the applied diffusion gradient and the primary diffusion direction of the tensor (different values are therefore possible along the *x*‐axis depending on the primary direction estimated by the model fit). Only points from the high‐angular‐resolution scan are shown for clarity, although all data were fitted to obtain directional estimates. The tensor‐ball and tensor‐sphere models produced similar fits to the gradient direction data with a jagged fit line due to signal variation in the secondary diffusion direction, while the Watson‐ball‐sphere model did not capture these variations as well, although neither the tensor nor Watson models captured all variation in the signal with gradient direction.

Similar trends were observed for the low‐SNR voxel (Figure 1C), but with smaller differences between the fits of the tensor‐ball and tensor‐sphere models and larger anisotropy.

**Figure 2:**
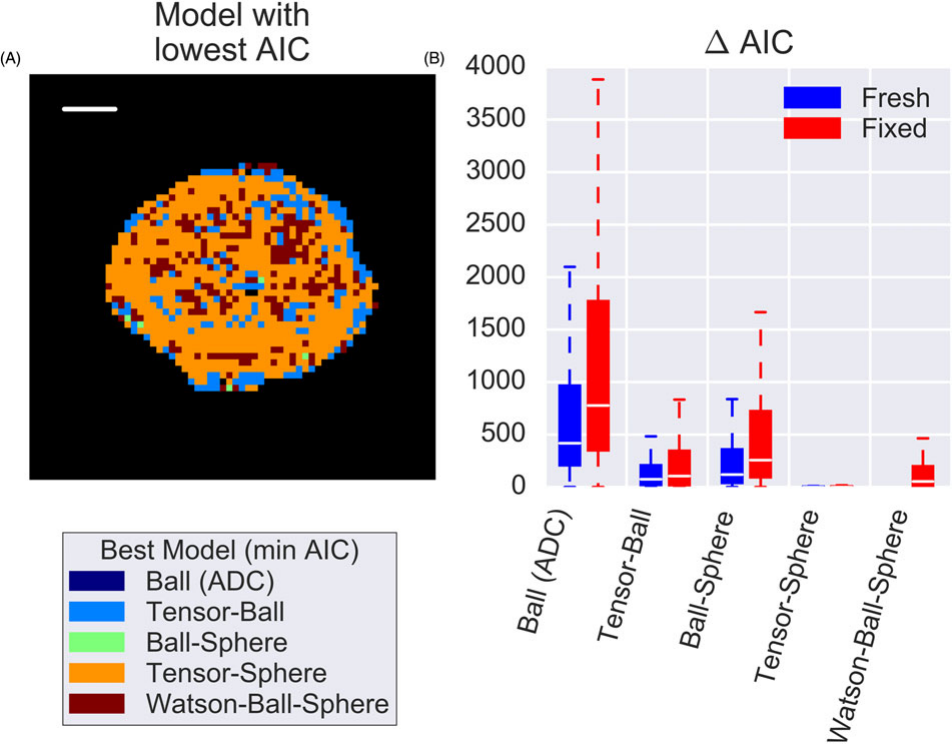
Model selection demonstrates that A, in this fixed sample, the tensor‐sphere model best described the data (lowest AIC) in most voxels. In some regions later found to contain more lumen space, simpler models were sufficient, but B, a boxplot of the relative AIC values confirms that, for most voxels in both fresh and fixed cases, the tensor‐sphere model explained the data better than a two‐compartment model without restriction (tensor‐ball) and a two‐compartment model without anisotropy (ball‐sphere)

Figure 2 demonstrates that the tensor‐sphere model had the lowest AIC in most voxels. A limited selection of models is presented to highlight the effects of incorporating restriction and anisotropy, but data from other models can be found in Supplementary Figures 2 and 3. To examine the effects of fixation on model parameters and compare with histology, we used the tensor‐sphere model since it best described the data in most voxels.

**Figure 3:**
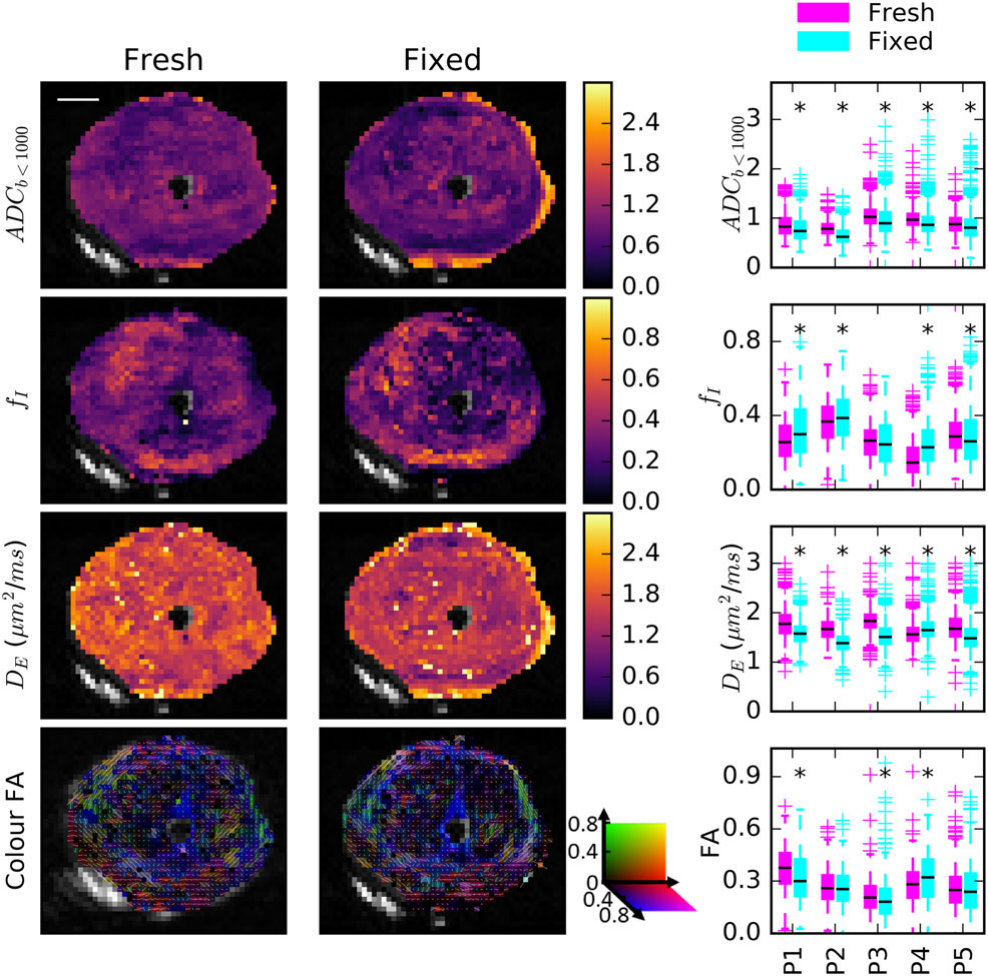
Comparison of the ADC_*b* < 1000_ calculation and selected tensor‐sphere model parameters in fresh and fixed samples. Parameter maps for a representative sample demonstrated similar spatial trends and absolute values before and after fixation. The boxplots summarize parameter values in all five samples. Black asterisks indicate that small but statistically significant decreases in ADC_*b* < 1000_ and *D*_E_ following fixation were the only consistent trends

Figure 3 shows selected parameter maps for one sample before and after fixation (see Supplementary Figure 4 for additional parameter maps). A map of ADC_*b* < 1000_ is presented for comparison with tensor‐sphere parameters. Although the ADC_*b* < 1000_ fits used fewer data, the results were similar to the ADC fit using all data. There was spatial heterogeneity in the maps but similar spatial patterns were observed before and after fixation with little change in the absolute values of the parameters, except in a few voxels of the fresh scan of Sample 2 where radius was poorly determined. These regions have low intracellular volume fraction and thus a small signal contribution from the restricted sphere component such that all values of *R* between the allowed fitting limits (0–20 μm) produce similar signal curves, making accurate determination of *R* challenging. The colour FA map in the bottom row demonstrates that the changes in directional parameters were also small.

The boxplots summarize the parameter changes in all five specimens and black asterisks indicate a statistically significant difference between fresh and fixed specimens. There was a small but significant decrease in ADC_*b* < 1000_ and *D*_E_ with fixation. Four of the five samples had increased *f*_I_ following fixation and this was due primarily to changes in the transition zone. The changes in radius, *R*, and FA with fixation were not significant in all specimens and did not show a consistent direction. Data are shown for the whole prostate, but tumour had significantly higher *f*_I_ and lower ADC_*b* < 1000_ than peripheral zone. The values of ADC_*b* < 1000_ across voxels had a strong correlation with *D*_E_ and strong inverse correlation with *f*_I_ from the tensor‐sphere model (see Supplementary Figure 5). ADC_*b* < 1000_ correlated weakly with *R*.

**Figure 4:**
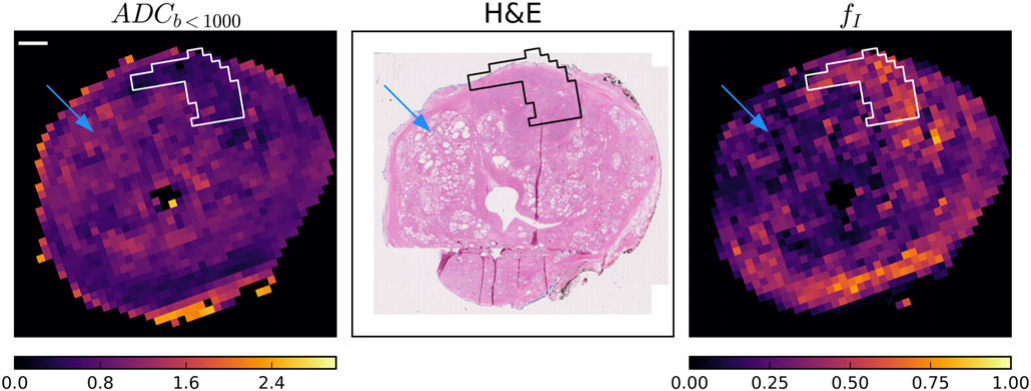
Representative ADC_*b* < 1000_ and *f*_I_ maps with registered H&E slice. The tumour region is in white on MRI and black on histology. Regions with substantial lumen space (blue arrows) correspond to regions with higher ADC_*b* < 1000_ and lower *f*_I_ in the MRI maps. However, ADC_*b* < 1000_ and *f*_I_ were also related to cell fraction, most clearly seen in the tumour regions, but in this case also near the bottom of the peripheral zone. Other specimen comparisons can be seen in supplementary figure 6. The scale bar is 10 mm

Figure 4 shows the ADC_*b* < 1000_ and *f*_I_ maps along with the registered histology reference slice in one of the five samples (remaining samples in Supplementary Figure 6). Regions of high *f*_I_ and low ADC correspond to regions of high cell fraction on histology, including in the Gleason 3 + 4 tumour outlined in black on histology. Regions of low *f*_I_ and high ADC are dominated by either lumen space (blue arrow) or low‐cellularity stroma. Some non‐cancerous regions of the transition zone also demonstrated higher *f*_I_ and these regions also appeared cellular on H&E.

**Figure 5:**
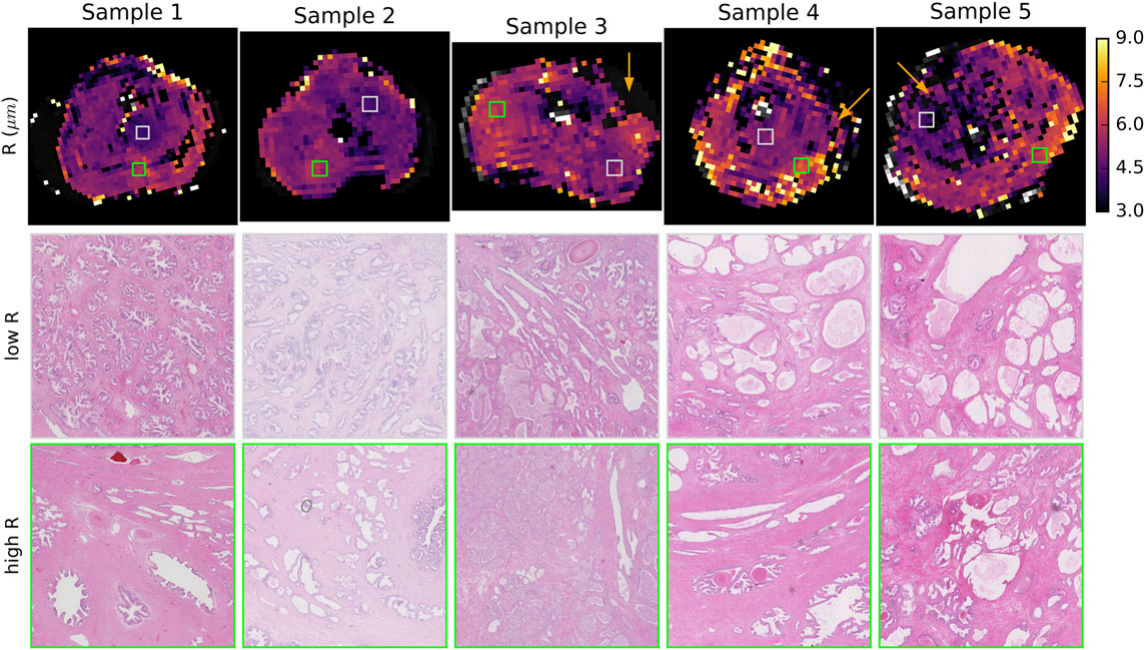
Comparison of *R* and cell size. Parametric maps of the VERDICT sphere radius *R* (top row) are relatively uniform with most values 6–7 μm, including in tumour regions. Orange arrows indicate several regions of low cellularity on histology where the sphere radius was poorly determined and thus is not plotted. White and green boxes indicate areas of low and high *R* that are depicted in high‐resolution histological images in the second and third rows

Figure 5 shows the maps of the sphere radius, *R*. In regions with substantial lumen space (orange arrows), *R* was poorly determined and tended toward the largest values allowed by the fitting (20 μm). These regions are not plotted in the *R* map because of the high uncertainty in the fitted parameter value. The remaining regions had a relatively uniform *R* indicating a sphere radius of about 6–7 μm. This was also the case in tumour regions (Samples 3 and 5). The white and green boxes highlight regions of lower and higher *R*, respectively, and the histology from these regions is outlined in the rows underneath each sample, but showed no obvious differences.

**Figure 6:**
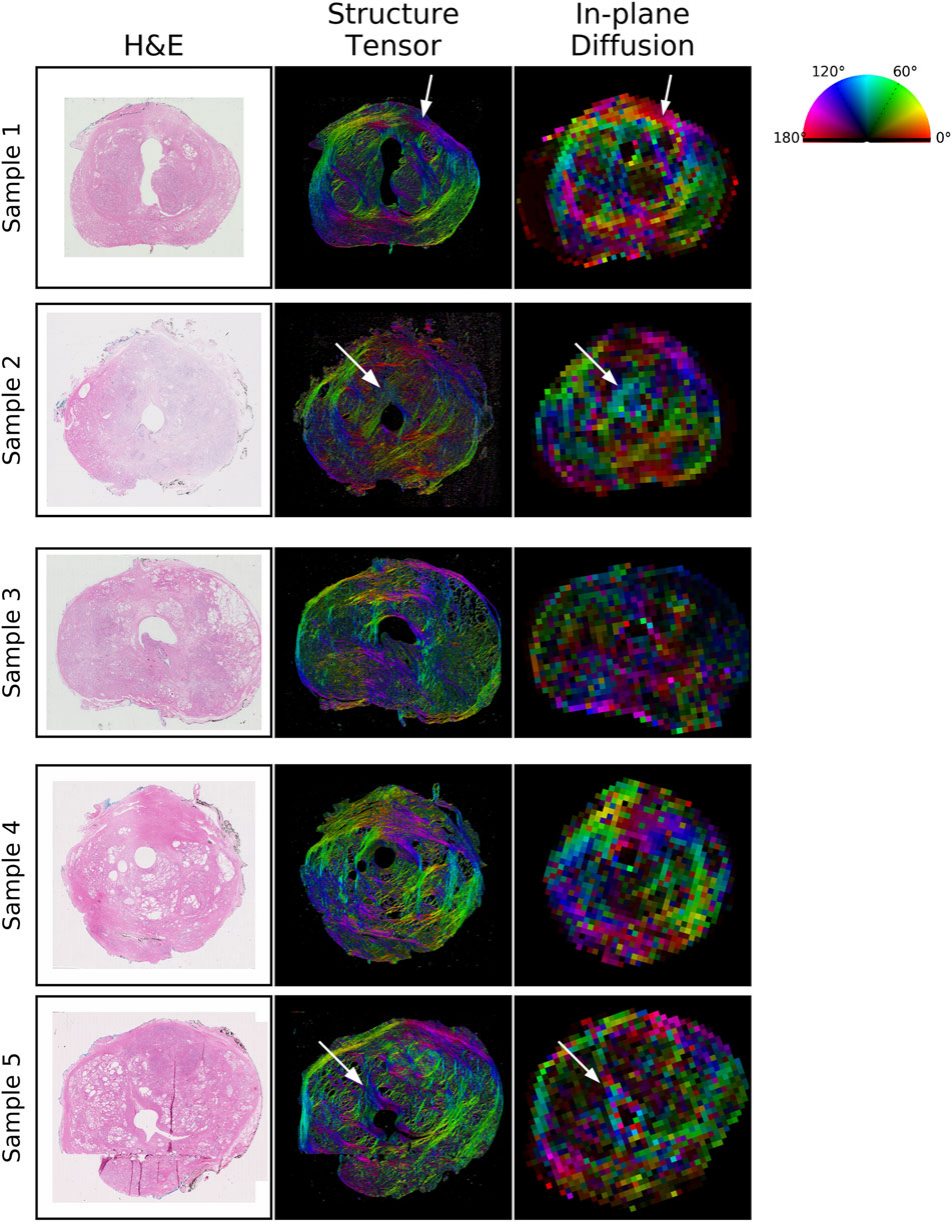
Anisotropy comparison for histology and VERDICT MRI. The first column shows the H&E histology image for each sample and the middle column is a structure tensor analysis of the stroma segmented from the histology, where colour indicates dominant fibre direction and brightness indicates the anisotropy index. For VERDICT analysis (right‐hand images), the colour represents the primary in‐plane diffusion direction and the brightness represents the 3D FA of the tensor component. White arrows indicate regions of bending or dispersing fibres described further in the text

The structure tensor analysis on the segmented stroma of the H&E image is shown in Figure 6 alongside the in‐plane colour FA map. In these maps, the direction is indicated by the colour in the legend, and these corresponded well in most regions of all samples. White arrows indicate several regions with a colour (directional) difference in regions of bending or dispersing fibres. The intensity corresponds to the anisotropy (anisotropy index for structure tensor and FA of the tensor compartment for MRI) and lower FA is observed in regions where the structure tensor suggests that there is less stroma or the direction in the stroma is less coherent over the MRI voxel scale.

## 4 DISCUSSION

This study implemented a patient‐personalized scanning and registration pipeline to validate VERDICT model parameters using histology. The rich diffusion data set allowed for examination of restricted and anisotropic signal components, including the novel testing of a Watson‐distributed sticks compartment in cancer. The mould limited tissue deformation and confined histological slicing to the MR imaging plane, allowing detailed histological comparison following rigid alignment with small residual errors due to slicing artefacts. In previous in vivo VERDICT studies, only biopsy data were available for parameter validation, with uncertainty due to biological heterogeneity and the spatial accuracy of biopsy sampling. The registered VERDICT maps and histological images confirmed previous in vivo VERDICT model findings, demonstrating high intracellular fraction in tumour regions.

### 4.1 Modelling water diffusion in prostate

The tensor‐sphere model best described the diffusion data in most regions of both fresh and fixed ex vivo prostate. This is in agreement with previous ex vivo studies.^7^ It also confirms in vivo findings that single‐compartment models such as the ADC or diffusion tensor fail when data include high *b*‐values or varying diffusion times,^8^, ^19^ but more complex models may capture additional information related to cellular fraction.

The sphere component captures restricted diffusion thought to be related to intracellular water; this was supported by the correspondence between the volume fraction of this compartment (*f*_I_) and the cellular fraction on histology (Figure 4). The SNR at high *b*‐values is also important for characterizing restricted diffusion (Figure 1C).

The tensor component of the model describes the anisotropic aspects of diffusion and corresponded to anisotropic features in the stroma (Figure 6). Although this anisotropic component has been observed in previous ex vivo studies,^7^, ^8^ it is not observed with in vivo VERDICT data. This is probably related to the coarser in‐plane resolution and more limited data. Although the model of Watson‐distributed sticks has been successfully employed to describe dispersing fibres in the brain,^10^ the Watson model did not describe signal variation with gradient direction as well as a tensor (Figure 1).

Other microstructural models, such as D‐Histo,^20^ also employ high *b*‐values to examine restricted diffusion in prostate cancer. The models differ in their approach to characterizing restriction. VERDICT estimates average cell size whereas D‐Histo separates signal into a highly restricted diffusion component related to lymphocytes and a restricted component related to epithelial cells. There were insufficient regions of inflammation in our data set to determine sensitivity to immune cell populations. In addition, VERDICT examines diffusion direction whereas D‐Histo analysis has focused on isotropic diffusion measures.

Alternative MRI contrast mechanisms also reveal microstructure. In luminal water imaging (LWI), the fraction of signal with long *T*_2_ relates to the lumen space and decreases in cancers as cells proliferate into the space.^21^ VERDICT does not distinguish extracellular water in the stroma from that in the lumen, while the *T*_2_ spectrum in LWI does not distinguish between non‐lumen water in the intracellular and stromal spaces. The two techniques are therefore likely to provide complementary information, but ex vivo LWI does not exhibit the same long‐*T*_2_ component.^22^ The combination of *T*_2_‐ and diffusion‐sensitive data is an important avenue for future in vivo studies. Indeed, hybrid multidimensional MRI,^23^ which explores *T*_2_ using a smaller range of echo times than LWI and lower *b*‐values than VERDICT, estimates lumen, epithelial and stromal fractions and correlates with Gleason score. Higher *b*‐values and techniques such as VERDICT that can characterize restricted diffusion may improve specificity further.

### 4.2 VERDICT model parameters—Cells

Variation of MRI parameters throughout the transition zone and peripheral zone appears to be true biological heterogeneity, with regions of lower cell density and more lumen space in Figure 4 exhibiting lower *f*_I_ and often higher ADC_*b* < 1000_. Variation in imaging properties throughout the prostate and its relation to biological variation has been noted previously.^24^

However, the higher intracellular fraction in tumour tissue relative to benign agrees with in vivo VERDICT findings^4^: Panagiotaki et al found median *f*_I_ ~ 0.5 in tumour and 0.2 in benign tissue. The volume fractions were also similar to those found in previous ex vivo VERDICT studies of fixed tissue.^7^

That *R* varies little between benign epithelial regions and tumour is not unexpected given that cancer cells are derived from epithelial cells. The *R* maps in a previous ex vivo VERDICT study showed more variation, including lower *R* values in non‐cancerous regions, but that study allowed the intracellular diffusion coefficient to vary during fitting, which can produce parameter couplings. The *R* range of about 6–7 μm found in this study corresponds to a cell diameter of 12–14 μm, in agreement with the value of 13.4 ± 2.5 μm found for cultured prostate cancer cells^25^ and just below the *R* of 8–8.5 μm in in vivo VERDICT studies,^4^ which may indicate some cell shrinkage after surgical removal.

The inverse relationship between *f*_I_ and ADC_*b* < 1000_, along with the corresponding histology, support the conventional wisdom that ADC relates to tumour cellularity. However, the data also demonstrated a weak correlation between ADC_*b* < 1000_ and *R*. A comparison of regions of low and high *R* in Figure 5 did not reveal obvious differences between these regions, and given the small number of samples further investigation is needed to interpret *R*. Nevertheless, the data suggest that ADC is related to intracellular fraction, but also influenced by other factors such as *R* that may affect its specificity as a biomarker.

### 4.3 VERDICT model parameters—Anisotropy

The relationship between diffusion anisotropy and extracellular features such as stroma and elongated stromal myocytes has been observed in previous work.^8^ The anisotropy is weaker than that observed in the white matter of the brain, but still visible, particularly when plotted using the tensor compartment's diffusion coefficients. The influence of stromal fibres and elongated myocytes on diffusion anisotropy is likely to be quite different from the restricted diffusion inside white matter axons, but similar errors in the directions obtained from the tensor model can be observed in areas where fibres change direction at a subvoxel scale (white arrows in Figure 6). For example, in Sample 1, fibres above the arrow's tip are curving slightly upward at an angle of about 160° (magenta‐blue colour), but fibres below the arrow's tip curve downward at an angle of about 20° (orange‐yellow). However, the MRI voxel corresponding to this area is red, corresponding to an angle of 0°, which would be the directional average of water influenced by all of these fibres.

In the brain and spinal cord, such fibre dispersion has been accounted for using the NODDI model,^10^, ^16^ which assumes a Watson distribution of fibres, but this model was not an improvement over the tensor model in prostate. However, given the failure of the tensor to characterize the primary diffusion direction in regions of dispersing and bending fibres, other fibre distributions or higher‐order spherical harmonics may better characterize anisotropic diffusion in prostate. Exploring such subvoxel variation in anisotropy is challenging with conventional diffusion techniques and new *b*‐tensor diffusion imaging and microscopic anisotropy techniques may offer better characterization of tissue microstructure.^26^, ^27^

Although FA in the tumour region itself has not demonstrated significant differences from benign tissue except in differentiating stromal benign prostatic hyperplasia,^28^ the role of diffusion anisotropy in non‐tumour tissue is relatively unexplored. Stroma has a role in cancer progression and invasion,^29^, ^30^ and non‐invasive biomarkers related to stromal features are therefore of interest. The colour FA maps plotted for VERDICT depict only the tensor component of the two‐compartment model, making it easier to see fibre direction in voxels with mixed cellular and stromal features than in colour FA maps derived from the conventional diffusion tensor.

### 4.4 Effects of fixation

Model selection was not affected by the fixation procedure, except in parts of the transition zone of Sample 2 that could be described by simpler models such as tensor‐ball. However, the parametric maps for the tensor‐sphere model in this sample were consistent with the changes seen in other samples, except that *R* was poorly determined. This suggests that the more limited data for fresh samples limited the accuracy of *R* in regions of low cell fraction.

The parametric maps demonstrated similar spatial trends before and after fixation, and comparison of the absolute values suggested that changes were small, with a decrease in *D*_E_ and an increase in *f*_I_ as the only consistent trends in VERDICT parameters. Significance may have been affected by residual errors in the registration and the heterogeneity within both the transition and peripheral zones.

Both the decrease in the extracellular diffusion coefficient, *D*_E_, and the increase in the intracellular water fraction, *f*_I_, are consistent with water loss from the extracellular space during fixation. The extracellular diffusion coefficient was fitted without a tortuosity approximation, so a decrease in the extracellular space is expected to increase tortuosity and decrease the diffusion coefficient, although there may also be some change in the intrinsic extracellular diffusion coefficient due to protein cross‐linking during fixation. The water fractions are relative, so an apparent increase in *f*_I_ is consistent with water loss if more water is lost from the extracellular than the intracellular compartment. The *f*_I_ increase was larger in the transition zone and the reason is unclear, but a comparison of the in vivo and ex vivo peripheral zone volumes suggests a collapse in the peripheral zone immediately after surgery, such that peripheral zone water loss may occur even before fixation. The change in the sphere radius *R* was not significant, which also suggests that more water leaves the lumen and extracellular spaces than the cells.

These changes with fixation are also consistent with previous ex vivo studies in prostate,^8^, ^31^ as well as brain^32^ and optic nerve,^33^ which suggest that fixation influences water content and diffusion coefficients, but neither model selection nor model parameters associated with tissue structure (*R*, directional patterns), are greatly affected by fixation.

These small changes suggest that fixation is unlikely to substantially affect estimates of VERDICT parameters related to tissue microstructure such as cell fraction, cell size or primary diffusion direction. A validation pipeline for in vivo VERDICT may therefore compare these parameters with histology without additional corrections for fixation.

### 4.5 Limitations

The models involve several assumptions and simplifications in order to make data fitting tractable. The *T*_2_ was modelled as a mono‐exponential decay, an assumption that was checked using a multi‐echo *T*_2_ sequence. This differs from the multi‐exponential *T*_2_ behaviour that has been observed in vivo,^21^ and *T*_2_ values were also shorter than those observed in vivo. This is probably related to differences in extracellular space, such as the collapse of some luminal spaces after surgery, water efflux and possibly increases in water exchange between the lumen and stromal spaces. Incorporating multi‐exponential *T*_2_ into a diffusion model is complex given that exchange is relatively fast compared with differences in *T*_2_ relaxation rates^34^ but slow for the majority of diffusion weightings and times.^35^ We therefore limited the scope of this paper to models with a single *T*_2_ component in all regions, which may have resulted in some systematic errors in regions with multi‐exponential *T*_2_ decay.

VERDICT models cell size with a single average radius, but cells have a distribution of sizes that can be captured by more complex models that assume a size distribution.^36^, ^37^ The amount of lumen space and the organization of cells around the lumen are also key features in histological grading. VERDICT currently does not distinguish extracellular water in the stroma from that in the lumen, but the directional diffusion findings in this study suggest this may also be possible by incorporating anisotropy information into future in vivo VERDICT work.

For model stability at low SNR, the intracellular diffusion coefficient, *D*_I_, was fixed to 0.3 × 10^−3^ mm^2^/s. This value was estimated from a fit to a high‐SNR region and it was assumed that intracellular diffusion was constant throughout the prostate. This value is in agreement with that from high‐resolution ex vivo studies of epithelial prostate regions.^9^ It has also been previously demonstrated that *D*_I_ has limited influence on the fit except at low radius values, where parameter coupling between *R* and *D*_I_ can occur.^13^

Comparison of MRI and histology was limited by the registration accuracy. The use of a rigid transformation simplifies registration but does not correct for all deformations between MRI and histology, particularly histological slicing artefacts near the urethra. Non‐rigid methods were unable to correct for these deformations. Differences in slice thickness and through‐plane location may also contribute to errors. Quantitative voxelwise comparisons should therefore involve caution, but the method is accurate enough for visual comparison and ROI‐based analysis.

Structure tensor analysis was subject to the 2D nature of the histological images. The mould allowed similar image planes so that most in‐plane directions should be comparable. However, when the primary diffusion direction is perpendicular to the image plane, the secondary diffusion direction may have a larger contribution to in‐plane diffusion. The secondary diffusion direction is not considered when constructing the in‐plane colour FA maps and comparison in these cases may be inaccurate. The use of the 3D FA to scale the image brightness will be most misleading in cases where the primary diffusion is through plane, which is expected to be a small number of voxels given a spherical probability distribution of angles.

Data were acquired from five samples, only two of which contained cancer in the reference slice. Four samples were Gleason 3 + 4 and one was Gleason 3 + 3. The small number of samples and limited range of Gleason grades mean that this study examines a limited subset of prostate cancers with moderately favourable outcomes. These specimens do not represent the full range of microstructures present in prostate tissue and cancers. In particular, cancers with Gleason scores lower than 3 + 4 may not show the same differences in VERDICT intracellular fraction, since there is less cell proliferation into the lumen space. Nonetheless, the patterns related to cellularity and fibre orientation were observed in both cancerous and non‐cancerous tissues and registration allowed for a detailed spatial comparison. The similarity in VERDICT parameters with fixation and registration of MRI to histology can be used as the basis for developing a full pipeline to validate in vivo VERDICT parameters. This study will involve a larger number of patients with a wide variety of prostate microstructure.

## 5 CONCLUSIONS

This study developed the ex vivo portion of a pipeline to validate VERDICT MRI in prostate using histology. The first part of the pipeline focused on fixation effects and demonstrated that VERDICT parameter changes were small. Slices for histology were then cut in the MR image plane, guided by a patient‐specific mould, and rigid registration was sufficient for ROI‐based comparison with histology. This demonstrated that regions of high intracellular fraction from VERDICT corresponded to regions of higher cell density in histology, including in tumours, and the primary diffusion direction related to stromal orientation patterns on histology.

## FUNDING INFORMATION

This work was supported by funding from EPSRC platform grant EP/M020533/1, the CRUK‐EPSRC Cancer Imaging Centre at KCL/UCL and the National Institute for Health Research University College London Hospitals Biomedical Research Centre. E.P. and C.B. were supported by EPSRC fellowship EP/N021967/1. R.B. received research funding from the Australian National Health and Medical Research Council (Grant 1026467). H.W. is supported by Prostate Cancer UK grant PG14–014, H.P. and E.J. by PG14‐018‐TR2. S.H. is funded by the Centre of Excellence (CEO13_2‐002).

## Supporting information

Supplementary Figure 1 A representative portion of (a) a haematoylin and eosin‐stained section, with (b) the segmentation of the stroma using k‐means clustering (stroma in blue, cell nuclei in red, other in cyan) and (c) the structure tensor analysis in the stromal regions, with direction indicated by the colours in the legend.
Supplementary Figure 2 Fits to the remaining models for the green voxel in Figure 1. Single‐compartment models and models without a restrictive sphere component failed to capture the signal at high b‐values and models without an anisotropic compartment were unable to capture the variation with gradient direction (plotted by cosβ)
Supplementary Figure 3 The fresh scan of one specimen demonstrates some regions where the Ball‐Sphere or Tensor‐Ball models best described the data.
Supplementary Figure 4 Comparison of the remaining parameters (not shown in Figure 3) for the Tensor‐Sphere model in Fresh and Fixed samples. Parameter maps demonstrate similar spatial trends and absolute values before and after fixation for a representative sample. Boxplots summarize parameter values in all five samples with black asterisks to indicate statistically significant changes in a region following fixation. Four out of five sample demonstrate small but significant decreases in the perpendicular diffusion coefficients of the Tensor compartment, D_⊥1_ and D_⊥2_, consistent with water efflux. Apparent changes in the radius may be due to more precise fitting in the richer fixed diffusion data set.
Supplementary Figure 5 Correlations between ADC_b < 1000_ and Tensor‐Sphere parameters in a representative sample demonstrated a strong inverse relationship with the intracellular fraction f_I_, a strong correlation with primary extracellular diffusion coefficient D_E_ and a weak correlation with R, regardless of the zone of the prostate (TZ = transition zone, PZ = peripheral zone, Tum = tumour). There is no relationship between ADC and directional parameters, such as θ or φ.
Supplementary Figure 6 Maps of ADC_b < 1000_ and f_I_ maps with registered H&E histology slices for the remaining samples. The tumour regions are in white on MRI and black on histology. Regions with substantial lumen space (blue arrows) correspond to regions with higher ADC_b < 1000_ and lower f_I_ in the MRI maps. However, ADC_b < 1000_ and f_I_ were also related to cell fraction, including in more cellular regions of the transition zone (red arrows). Scale bars are 10 mm
