# Supplementary material for: VERDICT MRI validation in fresh and fixed prostate specimens using patient‐specific moulds for histological and MR alignment
Source: NMR Biomed. 2019 Feb 19;32(5):e4073. doi: 10.1002/nbm.4073 (PMC6519204; doi:10.1002/nbm.4073)
Supplement: Supplementary file 1 — Supplementary Figure 1 A representative portion of (a) a haematoylin and eosin‐stained section, with (b) the segmentation of the stroma using k‐means clustering (stroma in blue, cell nuclei in red, other in cyan) and (c) the structure tensor analysis in the stromal regions, with direction indicated by the colours in the legend. Supplementary Figure 2 Fits to the remaining models for the green voxel in Figure 1. Single‐compartment models and models without a restrictive sphere component failed to capture the signal at high b‐values and models without an anisotropic compartment were unable to capture the variation with gradient direction (plotted by cosβ) Supplementary Figure 3 The fresh scan of one specimen demonstrates some regions where the Ball‐Sphere or Tensor‐Ball models best described the data. Supplementary Figure 4 Comparison of the remaining parameters (not shown in Figure 3) for the Tensor‐Sphere model in Fresh and Fixed samples. Parameter maps demonstrate similar spatial trends and absolute values before and after fixation for a representative sample. Boxplots summarize parameter values in all five samples with black asterisks to indicate statistically significant changes in a region following fixation. Four out of five sample demonstrate small but significant decreases in the perpendicular diffusion coefficients of the Tensor compartment, D⊥1 and D⊥2, consistent with water efflux. Apparent changes in the radius may be due to more precise fitting in the richer fixed diffusion data set. Supplementary Figure 5 Correlations between ADCb < 1000 and Tensor‐Sphere parameters in a representative sample demonstrated a strong inverse relationship with the intracellular fraction fI, a strong correlation with primary extracellular diffusion coefficient DE and a weak correlation with R, regardless of the zone of the prostate (TZ = transition zone, PZ = peripheral zone, Tum = tumour). There is no relationship between ADC and directional parameters, such as θ or φ. Supp [file NBM-32-e4073-s001.docx]

## Mathematical description of model compartments

Mathematical descriptions of the model components are presented here for the PGSE sequence. Compartments were assumed to have no exchange of water between them such that the total signal in a voxel, S, was the sum of all signal compartments, S_i_, weighted by their respective fractions, f_i_: $S=S_{0}e^{-TE/T2}\sum_{i} f_{i}S_{i}$, where S_0_ is the equilibrium signal intensity, TE is the echo time and T2 is the T2 relaxation time constant (S_0_ and T2 are fitted parameters in all models).

The Ball compartment is equivalent to the signal for Gaussian diffusion: $S_{Ball}=e^{-bD_{1}}$ with $b=\left( \gamma g\delta\right)^{2}\left( \Delta-\delta/3 \right)$, where γ is the gyromagnetic ratio, g is the gradient strength, δ is the gradient duration and Δ is the gradient separation.

The Zeppelin is the product of signal along the primary diffusion direction and the direction perpendicular to this: $S_{Zeppelin}=e^{-b{\cos^{2} \psi D}_{1}}e^{-b(1-\cos^{2} \psi)D_{2}}$, where ψ is the angle between the normalized gradient direction, $\hat{g}$, and the parallel diffusion direction, $\hat{n}$, defined in spherical co-ordinates with θ and φ: $\cos\psi=\hat{g}\cdot\hat{n}$, $\hat{n}=\left( \sin\theta\cos\varphi,\sin\theta\sin\varphi,\cos\theta\right)$

The Tensor signal is given by $S_{Tensor}=e^{-b{\cos^{2} \psi_{1}D}_{1}}e^{-b{\cos^{2} \psi_{2}D}_{2}}e^{-b{\cos^{2} \psi_{3}D}_{3}}$, where $\cos\psi_{i}$ is the angle between the gradient direction and *i*^th^ eigenvector from the diagonalization of the diffusion matrix such that $\hat{n}_{1}=\left( \sin\theta\cos\varphi,\sin\theta\sin\varphi,\cos\theta\right)$, $\hat{n}_{2}=\hat{k}\cos\alpha+\left( \hat{k}\times\hat{n}_{1} \right)\sin\alpha+\hat{n}_{1}\left( \hat{k}\cdot\hat{n}_{1} \right)\left( 1-\cos\alpha\right)$, $\hat{k}=\left( \sin\theta+\frac{\pi}{2}\cos\varphi,\sin\theta+\frac{\pi}{2}\sin\varphi,\cos\theta+\frac{\pi}{2} \right)$ is a vector orthogonal to $\hat{n}_{1}$ rotated by an angle α in that plane and therefore $\hat{n}_{3}\equiv\hat{n}_{1}\times\hat{n}_{2}$.

The Sphere signal is calculated using the GPD approximation (58): $S_{Sphere}=\exp\left( -2\gamma^{2}g^{2}\sum_{m} \frac{2{\beta_{m}}^{2}D_{1}\delta-2+2Y\left( \delta\right)+2Y\left( \Delta\right)-Y\left( \Delta-\delta\right)-Y(\Delta+\delta)}{{{{D_{1}}^{2}\beta}_{m}}^{6}\left( {\beta_{m}}^{2}R^{2}-2 \right)} \right)$. Here, $Y\left( x \right)=e^{-{\beta_{m}}^{2}D_{1}x}$, β_m_ is the m^th^ root of $J_{3/2}\left( \beta_{m}R \right)-\beta_{m}R J_{5/2}\left( \beta_{m}R \right)=0$ and J_ν_ is the Bessel function of the first kind, order ν. The summation was carried out over the first 31 roots of the equation.

The Watson compartment is a distribution of “Sticks”, where diffusion in sticks is restricted to a single dimension: $S_{Watson}=\int f(\hat{n})e^{-bD_{1}{(\hat{g}\cdot\hat{n})}^{2}}d\hat{n}$ where $\hat{n}$ is the direction of a single stick and their probability distribution $f\left( \hat{n} \right)=M\left( \frac{1}{2},\frac{3}{2},\kappa\right)^{-1}e^{\kappa{(\hat{\mu}\cdot\hat{n})}^{2}}$ where M is a confluent hypergeometric function, $\hat{\mu}$ is the mean orientation of the sticks and κ is the concentration parameter describing the extent of the dispersion.


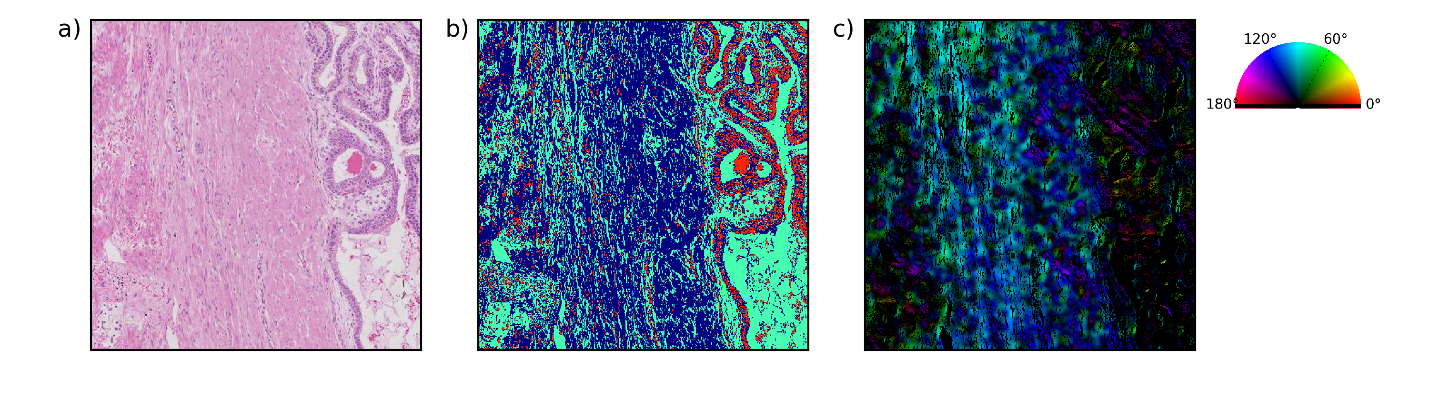


Supplementary Figure 1 A representative portion of (a) a haematoylin and eosin-stained section, with (b) the segmentation of the stroma using k-means clustering (stroma in blue, cell nuclei in red, other in cyan) and (c) the structure tensor analysis in the stromal regions, with direction indicated by the colours in the legend.


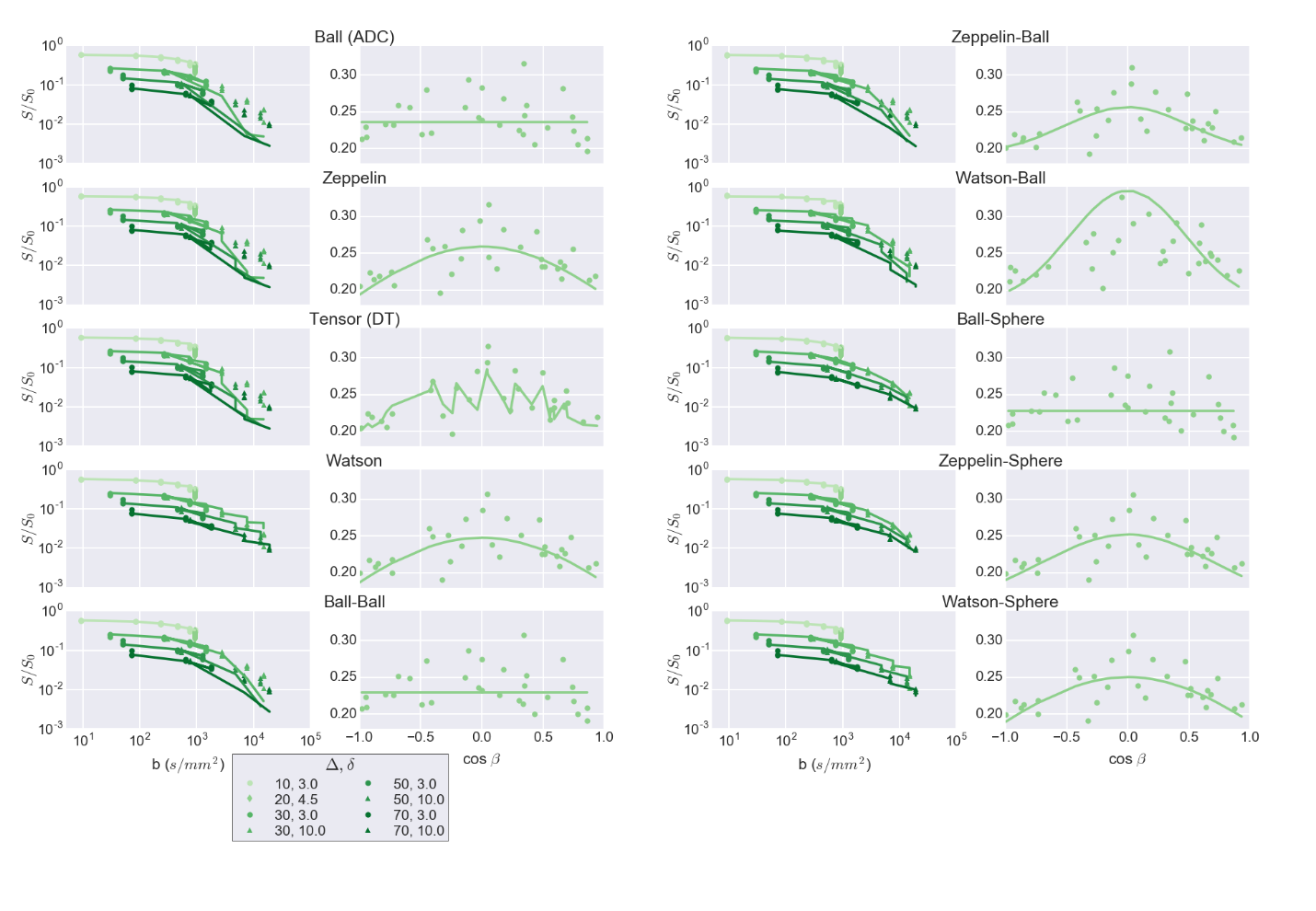


Supplementary Figure 2 Fits to the remaining models for the green voxel in Figure 1. Single-compartment models and models without a restrictive sphere component failed to capture the signal at high b-values and models without an anisotropic compartment were unable to capture the variation with gradient direction (plotted by cosβ)


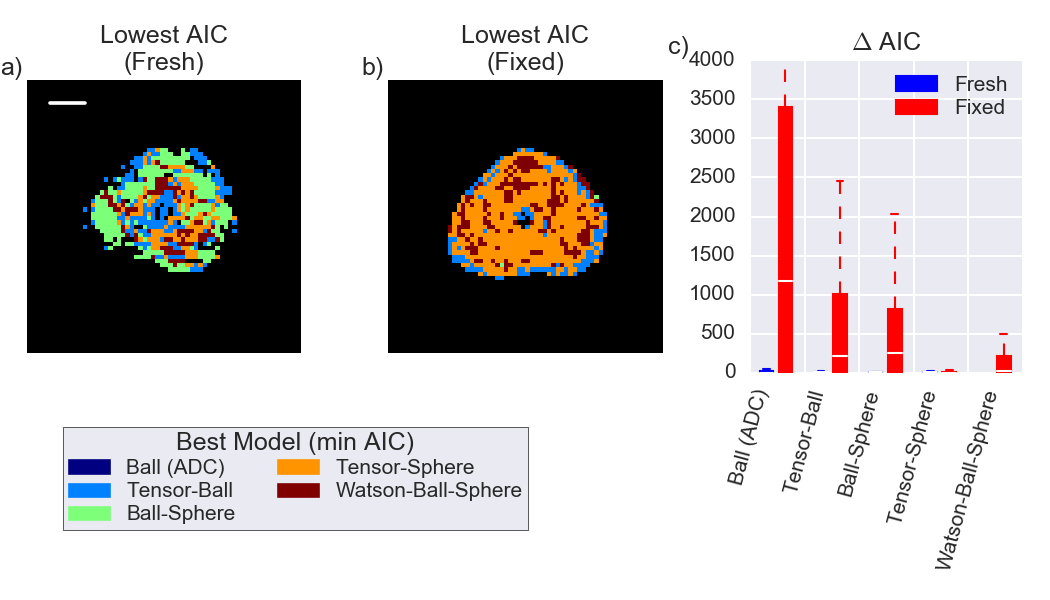


Supplementary Figure 3 The fresh scan of one specimen demonstrates some regions where the Ball-Sphere or Tensor-Ball models best described the data.


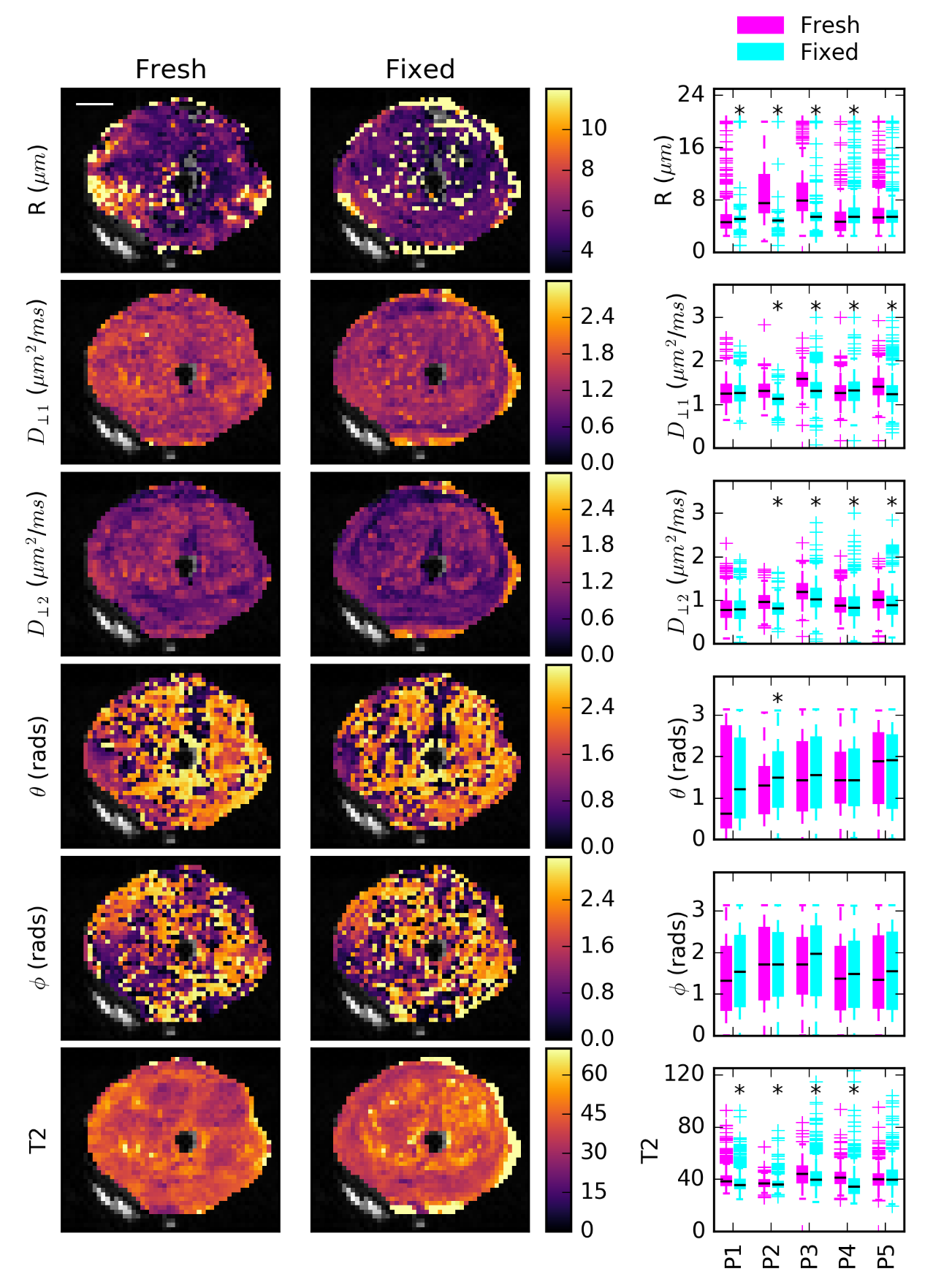


Supplementary Figure 4 Comparison of the remaining parameters (not shown in Figure 3) for the Tensor-Sphere model in Fresh and Fixed samples. Parameter maps demonstrate similar spatial trends and absolute values before and after fixation for a representative sample. Boxplots summarize parameter values in all five samples with black asterisks to indicate statistically significant changes in a region following fixation. Four out of five sample demonstrate small but significant decreases in the perpendicular diffusion coefficients of the Tensor compartment, D_⊥1_ and D_⊥2_, consistent with water efflux. Apparent changes in the radius may be due to more precise fitting in the richer fixed diffusion data set.


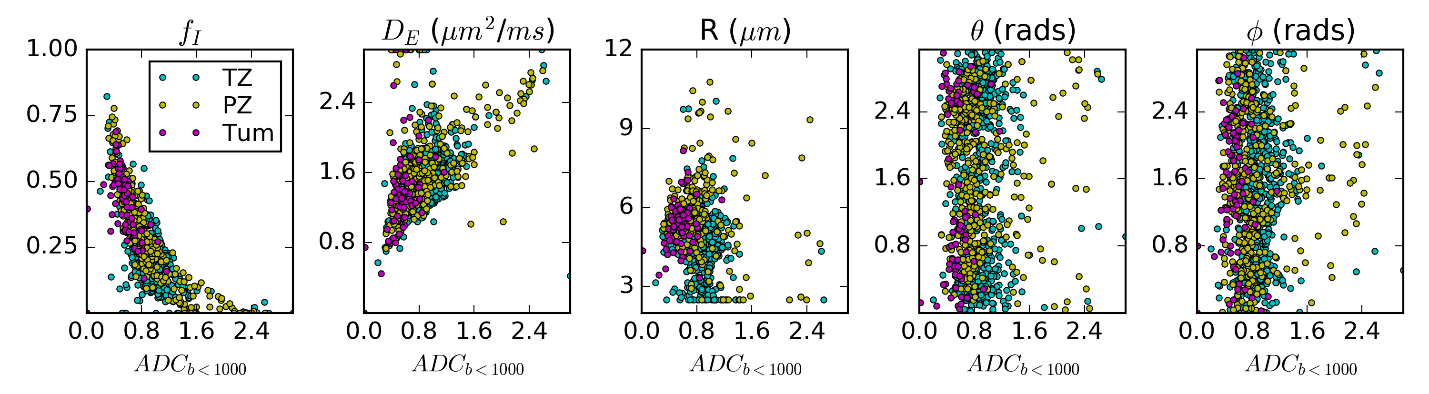


Supplementary Figure 5 Correlations between ADC_b<1000_ and Tensor-Sphere parameters in a representative sample demonstrated a strong inverse relationship with the intracellular fraction f_I_, a strong correlation with primary extracellular diffusion coefficient D_E_ and a weak correlation with R, regardless of the zone of the prostate (TZ=transition zone, PZ=peripheral zone, Tum=tumour). There is no relationship between ADC and directional parameters, such as θ or φ.


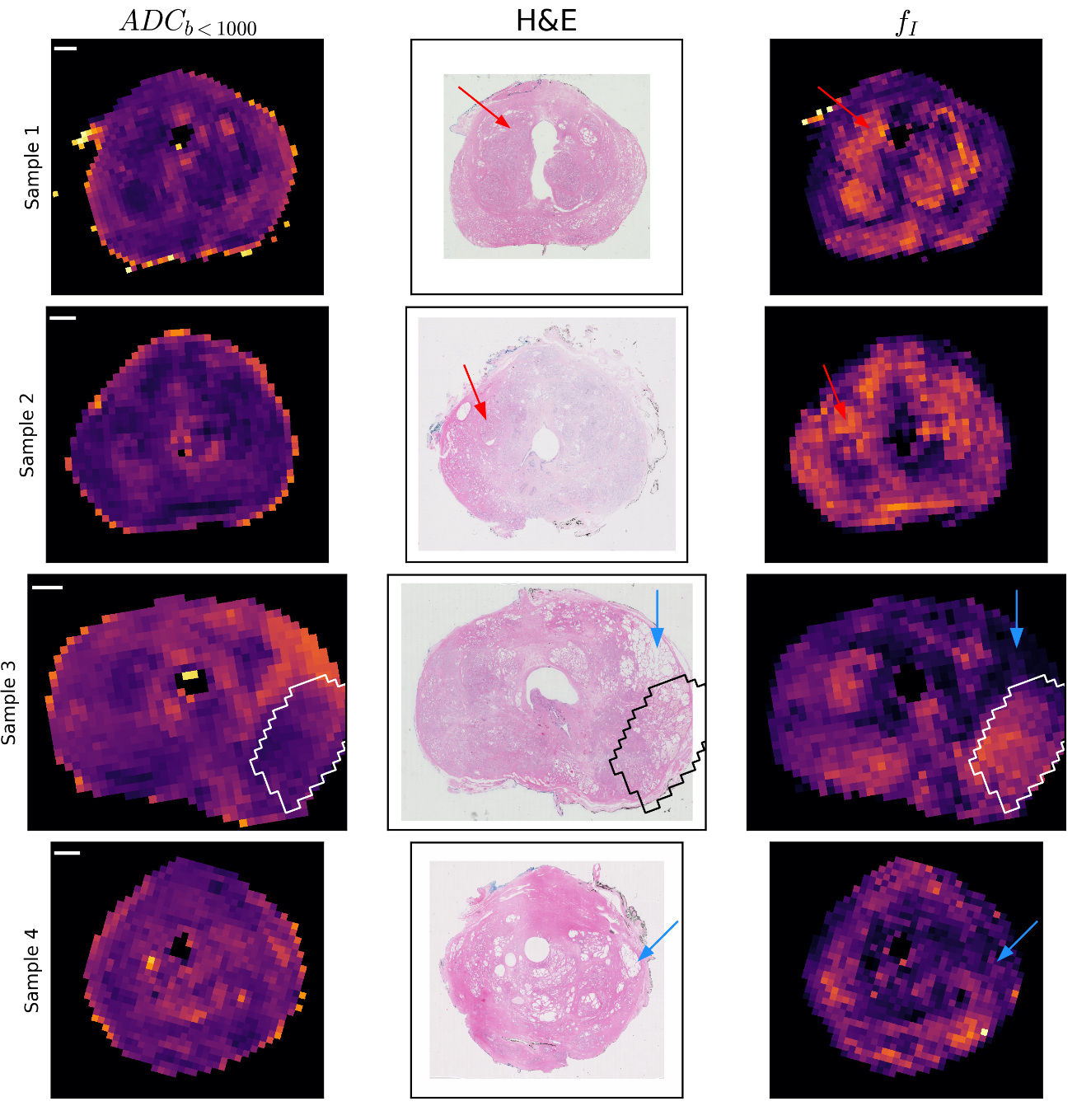


Supplementary Figure 6 Maps of ADC_b<1000_ and f_I_ maps with registered H&E histology slices for the remaining samples. The tumour regions are in white on MRI and black on histology. Regions with substantial lumen space (blue arrows) correspond to regions with higher ADC_b<1000_ and lower f_I_ in the MRI maps. However, ADC_b<1000_ and f_I_ were also related to cell fraction, including in more cellular regions of the transition zone (red arrows). Scale bars are 10 mm
